# Ætiology of Parenchymatous Nephritis

**Published:** 1859-03

**Authors:** 


### MEDICAL CLINIC.

### ÆTIOLOGY OF PARENCHYMATOUS NEPHRITIS.

From the "Allgemeine Medicinische Central Zeitung."

Dr. S. Rosenstein has become convinced, after a long series of observations, that the various symptoms which appear in Bright's disease are, in a very small number of cases only, evidence of primary lesion, but very much oftener the result of a peculiar diathesis, whose character is marked either by a mechanical obstruction of the circulation, or far more frequently by inaction in the function of nutrition and elimination, as also by diminution of the solid portions of the blood.

More especially has Dr. R. observed affections of the kidneys after intermittent fever, in which they appeared after long continued attacks, as results of an impoverished condition of the blood, following affections of the spleen, and also after depositions of coloring matter, the relation of which as a symptom of obstructed renal circulation has been shown by Frierich. Dr. R., however, considers the first cause as by far the most important, since cases of marked anæmia with less deposition of coloring matter, show clear lesions of the kidneys, while long continued albuminuria, associated, however, with unimportant anatomical changes of the epithelium, only are observed in melanæma. In those cases of intermittent, in which the perspiratory symptoms failed to appear in the paroxysm, the inaction of the skin was evidently of great significance, since diaphoretics often gave relief. The case next in importance was found in valvular disease of the heart, and diseases of the arterial walls, especially atheromatous formations, since these affections, unless their effects were counterbalanced by dilation and hypertrophy of the heart, produce passive and active renal hyperhæmia, which finally terminates in Bright's disease; yet there are cases in which the cause cannot be traced to mechanical obstruction.

Those conditions of the lungs also, in which there is an obstructed circulation, induce nervous renal hyperhæmia, and with it, temporary albuminuria, seldom, however, Bright's disease; and yet, Dr. R. would not cite these conditions as absolute causes, since eleven per cent. of the cases only, which fell under his observation, admitted of such an explanation. Ætiologically, and also numerically considered, those cases should be classified as tuberculous, in which long suppuration of the bones induces grave constitutional disturbance of nutrition, as a reflex of which we observed the renal affection. Certain surgical operations, particularly amputations, cause Bright's disease, not, as Siebert supposed, from any connection with lesions of the cuticular nerves, or with the situation (high or low) of the operation, but from some previous lesion, or from abnormal conditions developed after the amputation, as irritative fever, and suppuration, in the origin of which, loss of blood is of great influence. In pregnancy and the puerperal state, which give rise to renal difficulties, we should bear in mind the physiological impoverishment of the blood in solid portions, and also the mechanical obstruction of the renal veins. Next in frequency, Dr. R. observed the affection after cholera, typhus, and scarlatina, in each of which diseases it appears in five per cent. of the cases. In scarlatina the disease commences with capillary apoplexy and "croup" of the tubuli uriniferi. In typhus, the disease commences with catarrhal, and terminates with pseudo-membranous and parenchymatous inflammation, although the process terminates not unfrequently with simple catarrh. It commences at the end of the first fortnight, and is no serious prognostic symptom. In respect to the histology of the process, we must distinguish nephritis after scarlatina, from nephritis following typhus and cholera, a distinction based naturally upon the cause, since nephritis in these two affections is to be regarded as secondary, while in scarlatina it seems to be a local manifestation of the disease itself, as for example, ulceration of the bowels in typhus abdominalis. As in typhus, catarrhal becomes the cause of parenchymatous nephritis, in those cases, in which the excretion of urine is checked by catarrhal inflammation of the tubuli, so the same process is carried on (in the respective organs) in vesical catarrh, in strictures and in hypertrophy of the prostate.—*Virchow's Archiv. for Pathological Anatomy, Physiology, and Clinical Medicine,* xiv. 1, 2.

